# *Aggregatibacter actinomycetemcomitans* Leukotoxin Activates the NLRP3 Inflammasome and Cell-to-Cell Communication

**DOI:** 10.3390/pathogens11020159

**Published:** 2022-01-26

**Authors:** Peyman Kelk, Nick Sina Moghbel, Josefine Hirschfeld, Anders Johansson

**Affiliations:** 1 Department of Integrative Medical Biology, Umeå University, 901 85 Umeå, Sweden; peyman.kelk@umu.se; 2 Department of Odontology, Umeå University, 901 87 Umeå, Sweden; nisi0014@ad.umu.se; 3 Periodontal Research Group, Birmingham Dental School & Hospital, University of Birmingham, Birmingham B5 7EG, UK; j.hirschfeld@bham.ac.uk

**Keywords:** *Aggregatibacter actinomycetemcomitans*, leukotoxin, NLRP3 inflammasome, IL-1β, osteoclast differentiation, bone resorption, cell-to-cell communication

## Abstract

Carriers of highly leukotoxic genotypes of *Aggregatibacter actinomycetemcomitans* are at high risk for rapid degradation of tooth-supporting tissues. The leukotoxin (LtxA) expressed by this bacterium induces a rapid pro-inflammatory response in leukocytes that results in cell death. The aim of the present study was to increase the understanding of LtxA-induced leukocyte activation mechanisms and of possible associated osteoclast differentiation. The effect of LtxA on activation of the inflammasome complex was studied in THP-1 wild type and in NLRP3- and ASC knockout cells. Cell-to-cell communication was assessed by fluorescent parachute assays, and THP-1 differentiation into osteoclast-like cells was investigated microscopically. The results showed that LtxA induced inflammatory cell death, which involved activation of the NLRP3 inflammasome and gap junction cell-to-cell communication. THP-1 cells treated with lipopolysaccharide (LPS) and LtxA together differentiated into an osteoclast-like phenotype. Here, LPS prevented LtxA-mediated cell death but failed to induce osteoclast differentiation on its own. However, pit formation was not significantly enhanced by LtxA. We conclude that *A. actinomycetemcomitans* leukotoxicity mediates activation of the NLRP3 inflammasome and cell-to-cell communication in the induced pro-inflammatory cell death. In addition, LtxA stimulated differentiation towards osteoclasts-like cells in LPS-treated THP-1 cells.

## 1. Introduction

*Aggregatibacter actinomycetemcomitans* is a facultative anaerobic Gram-negative coccobacillus associated with periodontal disease in young individuals [1]. Rapid degradation of the tooth-supporting tissues is often seen in the vicinity of the *A. actinomycetemcomitans* infected gingival pocket [2]. A high intra-species genetic diversity has been shown, which has, during evolution, resulted in the generation of highly virulent as well as harmless variants of this bacterium [3]. The JP2 genotype of *A. actinomycetemcomitans*, which is characterized by a 540 base pair deletion in the promoter region of the leukotoxin (LtxA) gene operon, has attracted substantial interest [4].

This genotype features an enhanced expression of leukotoxin (LtxA) and significantly increases the risk for clinical attachment loss in colonized individuals [5,6]. LtxA specifically affects human leukocytes and requires interaction with the lymphocyte function-associated antigen 1 (LFA-1) on the outer membrane of the target cell for induction of leukocyte dysfunction [7,8,9].

Interestingly, human monocytes/macrophages have an enhanced sensitivity to LtxA and release substantial amounts of bioactive and pro-inflammatory interleukin(IL)-1β before the target cell undergoes pyroptosis [10]. It is well established that inflammasome activation results in the release of IL-1β, a molecule associated with many inflammatory diseases [11]. Vice versa, the rapid production of IL-1β and also of IL-18 indicate an activation of the intracellular inflammasome complex [12,13]. The assembly of this multimer complex is associated with the pathogenesis of several degenerative diseases, including periodontitis [14,15]. Pyroptosis and osteoclast differentiation are both cellular processes with a central role in the pathogenesis of periodontitis [16,17].

The inflammasome complex consists of three proteins: pro-caspase-1, Apoptosis-associated Speck-like protein containing a Caspase recruitment domain (ASC) and a third protein that varies depending on the stimulus and is named Nucleotide-binding oligomerization domain, Leucine-rich Repeat and Pyrin domain-containing (NLRPs) [18]. Pro-caspase-1 is cleaved in the inflammasome and executes the secretion of active IL-1β and IL-18 [19]. The effect of *A. actinomycetemcomitans* exposure on the expression of various NLRPs in human monocytes was studied, and a significantly increased expression of NLRP3 was observed [20]. Activation of the NLRP3 inflammasome was also shown to induce bone resorption [21,22].

Cell-to-cell communication is an important cellular event in the activation of pro-inflammatory responses in human monocytes/macrophages [23]. Blockage of cell-to-cell communication inhibits osteoclast differentiation and the initiation of bone resorption [24,25]. The carcinoma cell line THP-1 is a sensitive target for *A. actinomycetemcomitans* LtxA and can differentiate into functional osteoclasts with appropriate stimuli [12,26]. However, the role of LtxA in the induction of osteoclast differentiation is unknown. The aims of the present study were to examine the mechanisms by which LtxA may induce monocyte/macrophage activation and differentiation, as well as cell death.

## 2. Results

### 2.1. Characterization of the THP-1 Cells

**Figure 1:**
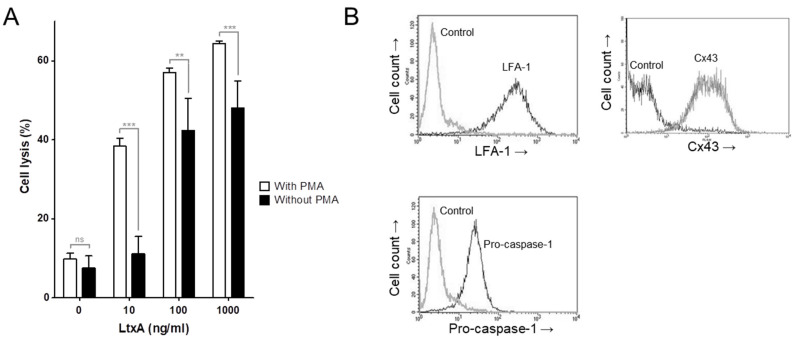
Characterizations of PMA stimulated and native THP-1 cells. (**A**) THP-1 cells, PMA stimulated or native, exposed to different concentrations of *A. actinomycetemcomitans* LtxA for 2 h. Leukotoxicity is expressed as the activity of LDH released from the damaged cells (cell lysis) compared to that of triton-lysed control cells (100%, not shown) (mean ± SD, *n* = 3 for with PMA and 5 for without PMA, ** *p* ≤ 0.01, *** *p* ≤ 0.001). (**B**) Expression of LFA-1, pro-caspase-1 and connexin 43 (Cx43) in native THP-1 cells. Right peaks represent fluorescence from cells labeled with the specific conjugated antibodies, and left peaks represent cells labeled with the corresponding conjugated isotype control. Representative results are shown.

The THP-1 cell is a human carcinoma monocytic cell line that, when stimulated with PMA, differentiates to an adherent macrophage-like phenotype. Both non-adherent native and PMA-stimulated THP-1 cells were sensitive targets for *A. actinomycetemcomitans* LtxA. Analysis of cell lysis was quantified by the release of lactate dehydrogenase (LDH), and THP-1 exposed to LtxA for 2 h showed a significantly enhanced sensitivity in the PMA-stimulated cell cultures (Figure 1A). Flow cytometric characterization of the native THP-1 cells showed a substantial expression of the LtxA receptor LFA-1, the channel protein connexin 43 (Cx43) (also known as gap junction alpha-1 protein) and the inflammasome protein pro-caspase-1 (Figure 1B).

### 2.2. Effect of Inflammasome Modifications on LtxA-Exposed THP-1 Cells

**Figure 2:**
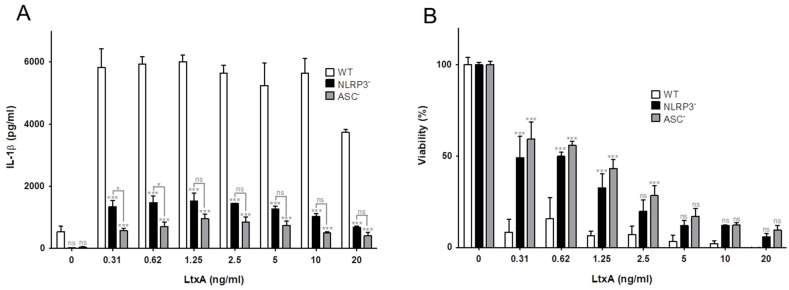
Effect of inflammasome modifications on LtxA-exposed THP-1 cells. PMA-stimulated THP-1 cells (wt, NLRP3^−^ or ASC^−^) were exposed to different concentrations of A. actinomycetemcomitans LtxA for 2 h. Significant differences between knockout cells and wt cells are indicated (* *p* ≤ 0.05. *** *p* ≤ 0.001). (**A**) IL-1β release into cell culture supernatants of the exposed cells determined by quantitative ELISA (mean ± SD, *n* = 3). Deletion of the inflammasome genes NLRP3 and ASC significantly inhibited LtxA-induced IL-1β release with significantly more inhibition in ASC^−^ cells at low LtxA concentrations. (**B**) Cell viability estimated by quantitative uptake of neutral red by the LtxA exposed cells (mean ± SD, *n* = 3). Deletion of the inflammasome genes NLRP3 and ASC significantly inhibited LtxA-induced cell lysis with no significant differences between the two modifications.

The PMA stimulated THP-1 cells responded to LtxA exposure with rapid activation and release of IL-1β (Figure 2A), followed by a decreased viability in the cell culture (Figure 2B). In order to determine the role of inflammasome activation in this activation, THP-1 cells lacking the genes for the inflammasome proteins, ASC or NLRP3, were used. These knockout cells were significantly less sensitive for the LtxA exposure compared with the wt cells (Figure 2). The ASC^−^ cells release significantly less IL-1β than the NLRP3^−^ at low LtxA concentrations.

### 2.3. Effect of LtxA in THP-1 Cell-to-Cell Communication

**Figure 3:**
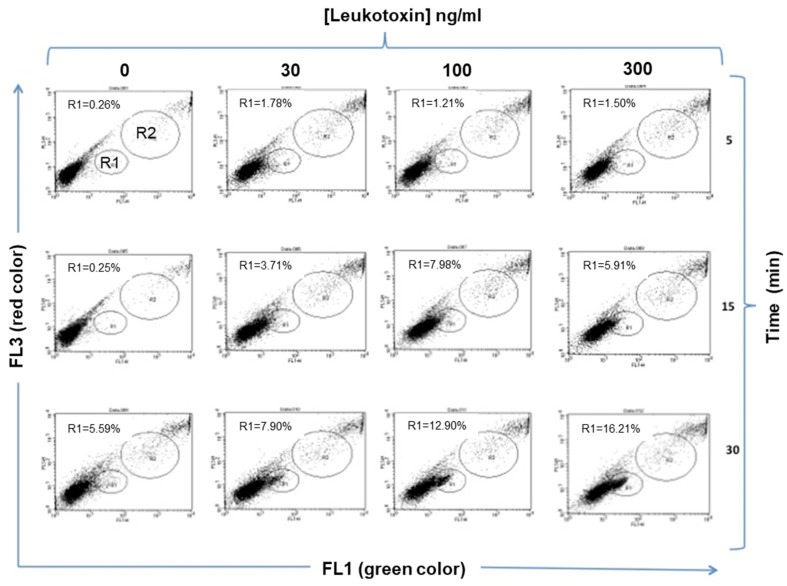
Effect of LtxA on THP-1 cell-to-cell communication. Time and dose screening of LtxA exposure on the activity of cell-to-cell communication in native THP-1 cells. The number of cells in R1 represents the recipient cells absorbing the fluorescent green color from the donor cells (upper right corner) when stimulated by different concentrations of LtxA (0, 30, 100, 300 ng/mL) for various times (5, 15, or 30 min). Cells in the lower-left corner are unstained recipient cells.

**Figure 4:**
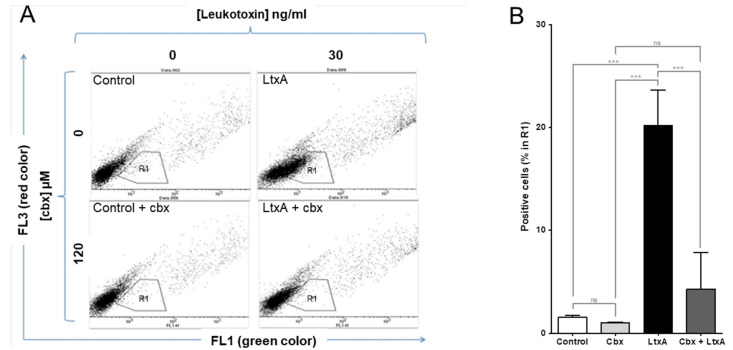
Effect of LtxA in THP-1 cell-to-cell communication. Native THP-1 cells were exposed to 0 ng/mL or 30 ng/mL of LtxA for 30 min ± 120 µM carbenoxolone (cbx). (**A**) Representative plots of LtxA-induced cell-to-cell communication analysed with the parachute technique. (**B**) Statistical data of cell-to-cell communication (mean ± SD, *n* = 3, *** *p* ≤ 0.001).

The parachute technique was used with double-stained native THP-1 cells as donor cells. The native THP-1 cells showed a dose- and time-dependent increase in gap junction cell-to-cell communication upon LtxA exposure (Figure 3), as analyzed in a screening with the parachute technique. The increased cell-to-cell communication induced by LtxA was efficiently (*p* < 0.001) blocked in the presence of the cell-to-cell communication inhibitor carbenoxolone (Figure 4).

### 2.4. Effect of Cell-to-Cell Communication in LtxA-Induced Primary Monocyte Activation

**Figure 5:**
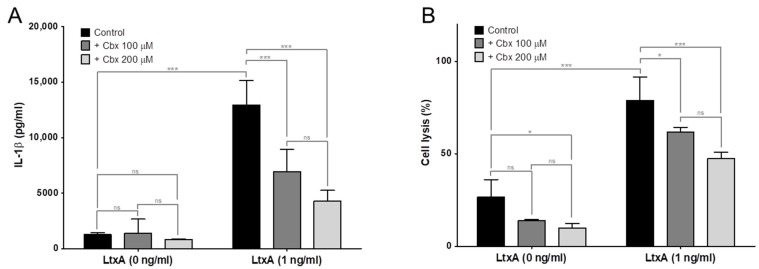
Effect of cell-to-cell communication in LtxA-induced monocyte activation. Human adherent blood-derived monocytes were exposed to LtxA (1 ng/mL) ± cbx (100 or 200 µM) for 2 h (mean ± SD, *n* = 6 for controls and 4 for exposed samples, * *p* ≤ 0.05. *** *p* ≤ 0.001). (**A**) LtxA induced IL-1β secretion determined in the cell culture supernatants by ELISA. (**B**) LtxA induced cell lysis determined as the activity of LDH released from the damaged adherent monocytes.

Results from LtxA-exposed THP-1 cells could be confirmed in cultures of adherent monocytes prepared from human peripheral blood. These cells responded to LtxA exposure by activation and secretion of IL-1β, followed by cytolysis, similar to that seen in the THP-1 cell line. These cellular events were significantly inhibited in the presence of cell-to-cell communication inhibitor carbenoxolone (cbx) (Figure 5).

### 2.5. Effect of LtxA in THP-1 Cell Differentiation into Osteoclast-like Cells and Bone Resorption

**Figure 6:**
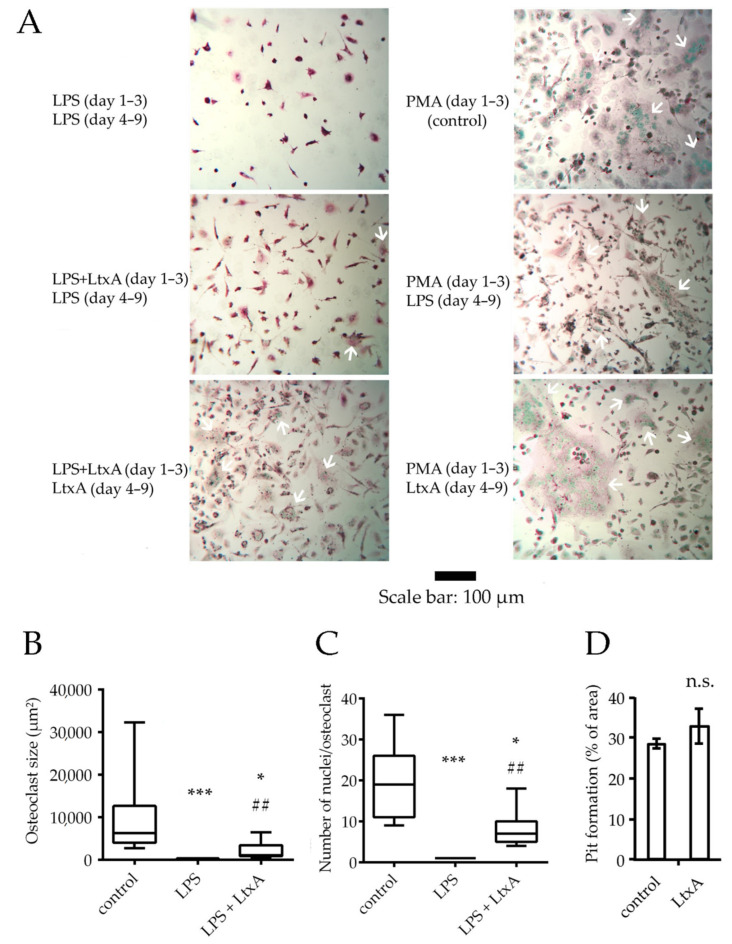
Osteoclast differentiation of THP-1 cells with and without LtxA. All cell cultures were differentiated with RANKL and α-tocopherol from day 4–9. LPS and/or LtxA were added as stimuli on days 1–3 and/or days 4–9. Control cells were differentiated with PMA (day 1–3) and with RANKL and α-tocopherol (day 4–9) (**A**) Microscopy images of cells differentiated under different conditions. Green: nuclei stained by methyl green, red: tartrate-resistant acid phosphatase (TRAP). White arrows: multinucleated cells. (**B**) The size of osteoclast-like cells and (**C**) number of nuclei seen per cell. LPS + LtxA: cells were incubated with both stimuli on days 1–3, followed by incubation with LtxA on days 4–9 in addition to RANKL and α-tocopherol. Kruskal–Wallis test, *** *p* ≤ 0.0001 and * *p* ≤ 0.05 compared to control, ^##^ *p* ≤ 0.01 compared to LPS (medians and ranges, *n* = 2 independent experiments in duplicate). (**D**) Pit formation ability of osteoclast differentiated THP-1 cells on 40 µm thick bovine cortical bone slices. Differentiation was achieved by exposing cells to PMA for 3d, followed by stimulation with RANKL, α-tocopherol with or without LtxA. Light microscopy was performed, and the surface area of pits in percent was determined by color filtration and binarisation. Two-tailed unpaired Student’s *t*-test, *p* = 0.23. (mean ± SD, *n* = 2 independent experiments in duplicate).

After 3 days, macrophage-like cells were observed in all experimental conditions (THP-1 cells stimulated with PMA, LPS or with LPS + LtxA). However, only a low number of cells became adherent and thus differentiated in the samples containing LPS alone. After a further 6 days of stimulation of macrophage-like cells with LPS or LtxA in addition to RANKL and *α-*tocopherol, large multinucleated cells were seen in all samples pre-differentiated with PMA. Moreover, such cells were seen in samples pre-differentiated with LPS and LtxA together and in those where LtxA was also added in the second stage of differentiation (Figure 6A–C). LPS alone failed to produce relevant numbers of osteoclast-like cells, as very few cells with more than two nuclei were seen. No significant differences in pit formation ability were observed between cells stimulated with LtxA and controls (Figure 6D).

### 2.6. Result Summary

LtxA from *A. actinomycetemcomitans* induces rapid pro-inflammatory cell death of human monocytes/macrophages in vitro. In the present study, we show that this activation involves the NLRP3 inflammasome and cell-to-cell communication. In addition, LtxA promotes differentiation in LPS-stimulated human macrophages (THP-1 cells) towards an osteoclast-like phenotype.

## 3. Discussion

In the present study, we investigated virulence mechanisms induced by *A. actinomycetemcomitans* LtxA that are associated with the enhanced bone-resorbing activity. Adolescents colonized with highly leukotoxic *A. actinomycetemcomitans* were shown to have a significantly increased risk of developing periodontal attachment loss [5,6]. It was previously reported that LtxA indirectly activates bone resorption by releasing substantial amounts of bioactive IL-1β [27]. Here, we used the THP-1 human monocyte cell line, which can be stimulated to differentiate into macrophage- or osteoclast-like cells [26,28]. In this research, we first confirmed that THP-1 cells are sensitive to LtxA and respond to LtxA exposure with pyroptosis [12]. This LtxA-induced activation is mediated by interactions with the outer membrane receptors LFA-1 and P2X_7_ [29], which are highly expressed by THP-1 cells [29,30]. Activation of the NLRP3 inflammasome is linked to inflammatory processes that are induced by a number of external factors, including bacterial toxins [31]. Observation from the present study showed a significant role of the NLRP3 inflammasome in the LtxA induced activation and killing of THP-1 cells.

LtxA exposure activated cell-to-cell communication in the THP-1 cell cultures, which could be inhibited by the connexin channel blocker cbx. A similar effect of LtxA on human lymphocytes was previously observed [9]. In addition, this inhibition also significantly decreased LtxA-induced IL-1β secretion and cell lysis. Therefore, these results indicate a new role of LtxA, activating cell-to-cell communication in human monocytes.

Our finding that LtxA activated the NLRP3 inflammasome and cell-to-cell communication prompted us to investigate a possible direct effect of LtxA on osteoclast precursors cells and on bone resorption. Both of these cellular events are crucial for osteoclast differentiation and activation [25]; moreover, blocking of the NLRP3 inflammasome as well as the connexin channels were shown to inhibit osteoclast differentiation [24,32]. Previous observations linked LtxA to bone resorption through a paracrine effect of the induced pro-inflammatory response [27], but the potential of LtxA to induce osteoclast differentiation directly has not been reported to date.

In the present study, we exposed THP-1 cells to both LtxA and LPS to investigate differentiation into osteoclasts. LPS was shown to strongly enhance osteoclast differentiation and bone resorption in RAW 264.7 macrophages [33], but not in THP-1 cells. Our research showed that in PMA-free samples, LPS alone failed to induce sufficient differentiation of THP-1 cells into macrophage-like cells, which is in accordance with previous reports from human monocytes [34], and consequently did not result in osteoclast-like cells. This finding is also consistent with reports that LPS stimulation leads to a transient attachment of THP-1 cells, followed by detachment [35].

Interestingly, the addition of LtxA enhanced attachment and differentiation of THP-1 cells at a concentration (50 ng/mL) that would normally lead to cell death in THP-1 cells and human monocytes [12]. When LtxA was added in the first and second phases of differentiation, osteoclast-like multinucleated cells were formed. The addition of LtxA only to the first phase (3d) of differentiation was not sufficient to yield relevant numbers of multinucleated osteoclasts after 9d.

These results indicate that LtxA is required for osteoclast differentiation in LPS-stimulated THP-1 cells. Vice versa, LPS appeared to counteract LtxA-induced THP-1 cell death, as no cell detachment was observed in these cell cultures. A possible underlying mechanism might be a competition for their common binding site, the beta-2 integrin subunit CD18 [36,37,38], which is also present on stimulated THP-1 cells [39,40]. This receptor competition could decrease the amount of membrane-bound LtxA and result in decreased leukotoxic activity.

Future research should be directed at revealing the mechanism by which LPS may protect THP-1 cells from LtxA-induced cell death and thus promote the cell adhesion seen in our assays. LtxA alone did not lead to significantly higher bone resorption than in the control. However, a possible synergistic or additive effect between LtxA and LPS as well as other bacterial products should also be investigated in future studies. In various host cells, both LPS and LtxA evoke the release of pro-inflammatory and pro-resorptive mediators [12,41]. Therefore, it is likely that multiple bacteria- and host-derived stimuli work together to enhance osteoclast activation in vivo.

Despite the clinical findings that strongly link the prevalence of highly leukotoxic *A. actinomycetemcomitans* with alveolar bone loss [5,6], a limitation of this study is the purely in vitro model. Whether the LtxA-induced cellular processes discovered in the present study play a critical role in vivo remains to be studied.

## 4. Materials and Methods

### 4.1. Cell Culture

The human acute monocytic leukemia cell line THP-1 (ATCC^®^ TIB-202^TM^) from the American Type Culture Collection (ATCC^®^, Manassas, VA, USA) was cultured in RPMI 1640 containing 10% fetal bovine serum (FBS) with a supplement of penicillin–streptomycin (Sigma Aldrich; St. Louis, MO, USA). Wild type (wt) THP-1 cells and the two THP-1 knockout cell lines NLRP3^−^ or ASC^−^ (InvivoGen, Toulouse, France) were used in some experiments. Thus, the gene deletion cell lines lacked key inflammasome components. The cells were incubated in a humidified atmosphere containing 5% CO_2_ at 37 °C. In the regular assays, the THP-1 cells were stimulated 24 h in the presence of 50 nM phorbol 12-myristate 13-acetate (PMA) followed by an additional 24 h period before the cells were exposed to different test stimuli. PMA stimulates differentiation of the non-adherent native monocyte-like THP-1 cell line into an adherent macrophage-like phenotype. In experimental set-ups that involved flow cytometric characterizations, the non-adherent native phenotype of the THP-1 cells was used.

### 4.2. Isolation of Human Monocytes from Peripheral Blood

Adherent monocytes were isolated from an enriched leukocyte fraction (buffy coat) of venous blood, as described previously [10]. Mononuclear leukocytes (MNLs) were isolated by isopycnic centrifugation in Lymphoprep (Nycomed AB, Lidingö, Sweden). The fraction containing MNLs was collected, and the cells were washed three times with phosphate-buffered saline (PBS) (250× *g*, 5 min) to remove platelets. The cell pellet was then re-suspended in culture medium RPMI-1640 containing L-glutamine, 10% fetal bovine serum (FBS), and penicillin–streptomycin (Sigma Aldrich) to yield 5 × 10^6^ cells/mL. This suspension was distributed into 50 mm Petri dishes (NUNC A/S, Roskilde, Denmark) at 15 mL/dish and incubated at 37 °C in 5% CO_2_ for 2 h to allow the monocytes to adhere. The non-adherent leukocytes were removed by two rinses with 10 mL PBS. The adherent cells were detached from the dish surface with a sterile cell scraper (Corning Incorporated, Corning, New York, NY, USA), washed twice with PBS and re-suspended in culture medium to yield 10^6^ cells/mL. Further, 1 mL of this suspension was distributed to 2 cm^2^ culture dishes (NUNC) and cultured for 20 h at 37 °C in 5% CO_2_ to equilibrate the cells. Before experimentation, the culture medium was replaced by a fresh medium. The MNLs obtained by this procedure are termed adherent monocytes.

### 4.3. Purification of A. actinomycetemcomitans LtxA

LtxA (leukotoxin) was purified from *A. actinomycetemcomitans* strain HK 1519 (JP2 genotype), described in detail previously [42]. The purified leukotoxin was basically free from LPS (<0.001% of total protein).

### 4.4. Flow Cytometric Characterization of the THP-1 Cells

The membrane expression of LFA-1 (CD11a, clone C38, IgG_2_) and Cx43 (clone 578618, IgG_2_) was detected with specific conjugated (FITC) antibodies (R&D Systems Inc., Minneapolis, MN, USA). The cell suspensions of native THP-1 cells were centrifuged for 300× *g* in 5 min, and the pellet was re-suspended in the antibody at 1% in PBS-BSA. The mixtures were incubated for 1 h before they were washed 3× with PBS and re-suspended in 500 µL PBS. Cells were analyzed in accordance with the manufacturer’s protocol in a FACS Calibur (Becton Dickinson Immunocytometry Systems, San Jose, CA, USA). For intracellular detection of pro-caspase-1, the cells were fixed and permeabilized using the Cytofix/Cytoperm™ kit (BD Biosciences Pharmingen, Eysins, Switzerland), and the protein was detected with a monoclonal antibody (14F468, IgG_1_, Bio-Techne Ltd., Abingdon, UK). Conjugated (FITC) isotype-specific antibodies were used for negative control cells (mouse IgG_2_ for CD11a and Cx43 and IgG_1_ for caspase-1). Ten thousand cells were analyzed for each marker in the flow cytometric detection, and all antibodies diluted 1:500 in the THP-1 cell suspensions (10^6^/mL). Stained cells were detected in the FL1 channel (530/30), and the THP—1 population was identified visually by forward and side scatter (FSC and SSC); debris and doublets were excluded.

### 4.5. Cytotoxicity Tests

The number of viable THP-1 cells in the culture was assayed at the end of the incubation by analyzing the uptake of neutral red dye [43]. For this purpose, the culture medium was replaced by a fresh medium containing 40 μg of neutral red/mL, and the incubation was continued at 37 °C for 90 min. The total amount of soluble dye in the extracts of the washed cells was measured in a spectrophotometer. Cell viability was expressed as the percentage of neutral red uptake found in the controls, i.e., cells not exposed to LtxA.

LtxA-induced cytolysis was determined as the release of the cytosol enzyme lactate dehydrogenase (LDH) into the culture medium as described earlier [10]. In brief, THP-1 cells were incubated for 2 h at 37 °C in the presence of LtxA, and the activity of the enzyme released from damaged cells into the supernatant was measured [44] and expressed as the percentage of the total LDH activity (100%) obtained by lysing the cells with 0.1% Triton X-100.

### 4.6. IL-1β Quantification

The amounts of IL-1β secreted into the culture medium of the THP-1 cells were determined by ELISA using DuoSet kits (R&D Systems Inc., Minneapolis, MN, USA). The ELISA test procedures were performed according to the manufacturer’s protocol. Previous observations showed the specificity of this kit for the 17 kDa active form of this cytokine [12].

### 4.7. Cell-to-Cell Communication Assay

Cell-to-cell communication was quantified using the fluorescent dye transfer parachute technique described by Czyz et al., 2000 [45]. Briefly, the donor cells (native THP-1 cells) were prepared with a staining solution using two dyes: the membrane-permeable green dye calcein-AM (1 μg; Molecular Probes Inc., Eugene, OR, USA), and the lipophilic red dye Dil (1 mM; Molecular Probes), used at final concentrations of 2 μM and 10 mM, respectively, for 30 min at 37 °C. The stained cells were washed with PBS and centrifuged three times (200× *g* for 5 min), and thereafter re-suspended in 1 mL PBS with 2% FBS. The double-stained donor cells were added to the unstained recipient cells at a ratio of 1:50 (donor:recipient) and incubated at 37 °C in 5% CO_2_ for 1, 2 and 3 h. The non-fluorescent dye calcein-AM is hydrolyzed by intracellular esterase’s into the fluorescent calcein and can, thereafter, only pass from donor to recipient cells through functional gap junction channels. The dye transfer was detected by flow cytometry, where recipient cells within the gated area showed a positive calcein signal. Donor cells were detected as cells with high intensities for both. Dil-stained cells in Channel FL3 (Ex/Em, 670LP) and calcein-AM-stained cells I channel FL1 (Ex/Em, 630/30). The gating strategy is shown in Figure 3. carbenoxolone (cbx) (Sigma Aldrich) was used as an inhibitor of cell-to-cell communication.

### 4.8. Osteoclast Differentiation

THP-1 cells were maintained at 2 × 10^5^ cells/mL in RPMI 1640 medium supplemented with 10% FCS and 2 mmol/L L-glutamine. THP-1 cells (2 × 10^5^/mL) were differentiated into macrophage-like cells on glass coverslips in 24-well plates for the first 3d (first stage of differentiation) using 10 ng/mL phorbol 12-myristate 13-acetate (PMA, Sigma-Aldrich, St. Louis, MO, USA), or 100 ng/mL LPS from Escherichia coli (Sigma Aldrich, Gillingham, UK) with or without 50 ng/mL LtxA. LtxA alone was found to lead to rapid cell death in THP-1 cells (Figure 2B) and was therefore not used as a sole stimulus. Next, these cells were incubated for 6 days (second stage of differentiation) in fresh RPMI 1640 (10% FCS, 1% L-glutamine) with 20 ng/mL RANKL (Novus Biologicals, Abingdon, UK) and 20 μM *α-*tocopherol (Sigma Aldrich), with or without 100 ng/mL LPS from *Escherichia coli* (*E. coli* O111:B4, Sigma Aldrich), 50 ng/mL LtxA or a combination of these stimuli. All samples were supplied with fresh media every 2d. TRAP staining was carried out using a TRACP and ALP double-stain assay kit according to the manufacturer’s instructions (Takara Bio Inc., Saint-Germain-en-Laye, France). Cells were considered osteoclasts if at least 3 nuclei and positive TRAP staining were seen under a light microscope. Osteoclasts were further characterized by image analysis using Image J (Version 1.8.0, NIH, Bethesda, Rockville, MD, USA). Cell sizes and numbers of nuclei of the 15 largest osteoclasts per area were evaluated at 10× magnification on 2 images.

### 4.9. Bone Resorption Assay

For pit formation assays, cells were differentiated on bone slices in 24-well plates. Each well contained a bovine cortical bone slice of 40 μm thickness. These were prepared using an IsoMet^TM^ low-speed bone saw (Buehler, Leinfelden-Echterdingen, Germany) with water cooling and were subsequently UV-sterilized. All experiments were performed in duplicate and according to the method previously described [46]. In brief, after differentiation of THP-1 cells as described in Section 4.8, bone slices were sonicated for 15 min in distilled water at 50 Hz in a sonication bath. Next, bone slices were fixed with 2.5% glutaraldehyde in PBS for 30 min at RT and stained with 1% toluidine blue in 1% sodium borate for 5 min. Light microscopy was performed at a 5× magnification. Pits were shown as purple-stained areas on the bone slices. From each bone slice in duplicate, 3 different areas were photographed, and color filtration to exclude all unstained areas was performed with Adobe Photoshop CS3 (Adobe Inc., San Jose, CA, USA). The images were then binarized using ImageJ, and the resorbed area percentage was determined. Osteoclast differentiation and bone resorption assays were performed at the Birmingham Dental School and Hospital, UK.

### 4.10. Statistics

Statistical significance was estimated by one-way ANOVA analysis with Bonferroni correction in GraphPad Prism version 9 (Figure 1, Figure 2, Figure 4 and Figure 5). The data presented in Figure 6 were analyzed as follows: two-tailed unpaired Student’s *t*-test was used for parametric data, and Kruskal–Wallis test for non-parametric data as determined by Shapiro–Wilk test. The statistical levels are indicated as * *p* ≤ 0.05, ** *p* ≤ 0.01 or *** *p* ≤ 0.001.

## 5. Conclusions

Inflammatory bone resorption is a key pathomechanism in periodontitis and is induced by the host response against invading bacteria. Carriers of highly leukotoxic genotypes of *A. actinomycetemcomitans* constitute a high-risk group for early onset of periodontitis. Here we show a new role of LtxA in activating osteoclast precursors. Both the LtxA molecule and the host response induced by this toxin are, therefore, potential targets for future therapeutic strategies. The compounds inhibiting leukotoxicity, the NLRP3 inflammasome or cell-to-cell communication, are well-known in the literature and could be explored regarding their application in the treatment of *A. actinomycetemcomitans*-mediated periodontitis.

## Data Availability

The data presented in this study are available on reasonable request from the corresponding author.
